# The Effectiveness of Written Asthma Action Plan at the National Guard Health Affairs' Asthma Clinic

**DOI:** 10.7759/cureus.6247

**Published:** 2019-11-27

**Authors:** Loie Goronfolah, Alwalla Abulaban, Ahlam I Barnawi, Maram Jawi, Wejdan Alhadhrami, Nada Y Baatiah

**Affiliations:** 1 Allergy and Immunology, King Abdulaziz Medical City, Jeddah, SAU; 2 Respiratory Therapy, College of Applied Medical Sciences, King Saud Bin Abdulaziz University for Health Sciences, Jeddah, SAU; 3 Clinical Nutrition, College of Applied Medical Sciences, King Saud Bin Abdulaziz University for Health Sciences, Jeddah, SAU

**Keywords:** asthma, chronic pulmonary disease, airway obstruction, airway inflammation, awareness, knowledge, quality of life, exacerbation

## Abstract

Introduction

Bronchial asthma has grown in epidemic proportions, and it is one of the most chronic diseases that affect many societies in the world. As managing asthma is complex, new management approaches have been developed, such as the written asthma action plan. This study aims to assign a baseline for the patients’ knowledge about asthma and its management and to assess their need for an asthma action plan. Then, to identify the effect of the written asthma action plan on the following parameters: exacerbations rate, and the frequency of using rescue mediations. Also, to compare the quality of life, functional limitations, and the level of patients’ self-confidence in treating their asthma before and after using the written asthma action plan.

Method

This study is a cross-sectional and interventional mixed-method study design. It was conducted at the National Guard Health Affairs (NGHA) asthma clinics between October 2017 to November 2017. Asthmatic patients who were above five years old and had no other lung comorbidities were evaluated before and after following the written asthma action plan by using three previously validated and published surveys that consist of five sections: demography, knowledge, quality of life, exacerbation rate, and overall evaluation.

Results

This study enrolled 58% (209) males and 42% (154) females. Regarding asthma medication knowledge and attack management, 62.3% of the patients do not adhere to their maintenance medications when they do not have asthma symptoms. Also, only 24.9% were very confident about knowing how to use their inhalers. For the impact of asthma on patients’ quality of life and the functional limitations, we found that 42.1% of patients were absent from school or work more than once a week because of asthma. While 61.0% of patients did not attend social events twice or less per week because of their asthma. The third section of the survey was about asthma exacerbation related events during the past year, we found that 39.0% of patients had one or more asthma attacks, 41.6% visited the emergency room (ER) once or more during the past year, and 28.1% of patients have been hospitalized because of their asthma. Finally, the section about patients’ evaluation of their asthma and their confidence about managing their condition, we found that around 20% of patients had poor or no control over their condition.

In the second phase of the study, which includes 60 subjects, we found that following the action plan helps in increasing the patients’ knowledge about their condition, and improves their quality of life and functional limitations as they learned how to cope with their symptoms. In addition, it has increased the confidence level of patients about controlling their asthma and decreasing the asthma exacerbation related events rates. Overall, the patients’ evaluation of their asthma has been increased significantly (p-value= 0.023).

Conclusion

Most of the asthmatic patients had insufficient knowledge and/or poor adherence to their treatment which impacted their quality of life. The written asthma action plan was effective in increasing the patients’ knowledge about their condition, improving their quality of life and functional limitations, and increasing their confidence level about controlling their asthma.

## Introduction

Asthma is a chronic pulmonary disease that is characterized by obstructed airways and marked by attacks of breathlessness and airway inflammation. Bronchial asthma has grown in epidemic proportions, and it is one of the most chronic diseases that affect many societies in the world [1]. Asthma affects a person’s daily life activity, including the physiological, social, and financial aspects. According to a study conducted in Saudi Arabia, the prevalence of asthma had increased by 15% between 1986 to 1995 due to the high exposure to environmental factors such as tobacco smoke and pets [2]. In the past few years, another study was conducted, and it showed that the prevalence of asthma increased significantly to reach more than two million Saudis. This significant increase suggests the lack of awareness of this disease among Saudi people and leads up to attract healthcare systems’ attention to the importance of asthma management. Structured questionnaires were randomly distributed in Saudi Arabia and demonstrated that “bronchial asthma knowledge in the Saudi Arabian population is insufficient, and efforts should be carried out to spread bronchial asthma management.” [3].

One of the most important efforts that have been done to support the management of asthma was initiating specialized asthma clinics which developed management programs to be followed instead of visiting the regular outpatient clinics. Asthma clinics have been proven to be more effective by a retrospective study conducted in the US, which compared asthma patients who are following an asthma clinic program with those who are seen in the primary care clinics. The results showed that following an asthma clinic program has helped in the reduction of emergency room (ER) visits and other asthma-related health costs [4-5]. These clinics promote asthmatic patients’ follow-up, meet their needs, improve patient education, and increase treatment compliance. Asthma clinics handle all types of asthma, whether it is mild, moderate, severe, allergic or non-allergic, controlled, or uncontrolled. Based on a study involved moderate-to-severe asthmatic patients, an improvement has been noted in the patients’ clinical parameters and several controlled asthma cases [6]. Furthermore, asthma clinics offer different approaches to help patients understand their roles in the management of their condition, one example of an approach done by the asthma clinic is a written asthma action plan.

An asthma action plan is an interventional tool that helps patients to control their condition; recognize exacerbations, and cope with their symptoms. It is a group of individualized instructions made after a discussion is carried out between the clinician and the patient about the proper way to use the medications according to the patient’s asthma level. As it is described in The National Asthma Council Australia (n.d.), asthma action plan follows a traffic light system for identifying the severity of exacerbation, which is moving from green (under control) to red (emergency) [7]. According to the National Asthma Council Australia (n.d.), all action plans should contain information about the proper action for each level of exacerbation, and cover the doses and frequencies of maintenance and rescue medications in response to particular signs and symptoms. Also, some essential details must be mentioned, such as the date, the patient’s name, and their doctor’s contact information. According to Pinnock (2015), “A crucial component of effective self- management interventions is the provision of an agreed, written action plan.” However, a Cochrane systematic review was published in 2003 on the Primary Care Respiratory Journal. It concludes that no enough evidence was found about the effectiveness of written asthma action plans in the improvement of asthma management in comparison to no written asthma action layouts [8]. Therefore, this study aims to determine the effectiveness of the written asthma action plan at the National Guard Health Affairs' (NGHA) Asthma Clinic.

## Materials and methods

This study is a cross-sectional and interventional mixed-method study design. The first phase of the study is an observational descriptive cross-sectional that aimed to assign a baseline for the patients’ knowledge about the disease and its management then to assess their need for an asthma action plan. A total of 363 participants who were above five years old and willing to participate in the study were included. Subjects who had other comorbidities such as interstitial lung disease, cystic fibrosis, bronchiectasis, and any chronic lung disease were excluded. The data were collected consecutively by interviewing (face-to-face or by phone) asthmatic patients who attended NGHA asthma clinics at Jeddah between October 2017 to November 2017.

To achieve our aim, three previously validated and published surveys were combined and modified in one structured Arabic survey consisting of five sections: demography, knowledge, quality of life, exacerbation rate, and overall evaluation [9-10]. The patients' demography consisted of age and gender. Followed by four questions that assess patients’ knowledge about asthma management and medications. Then four other questions to evaluate the impact of asthma on patients’ quality of life and functional limitations. The exacerbation section had questions about the rate of exacerbations, using rescue mediations, hospitalizations, and ER visits during the last year. Finally, the survey ends with two questions about patients’ overall evaluation regarding their asthma, and (if applicable) peak expiratory flow values.

In the second phase, prospective, interventional, the same asthmatic patients were invited and provided/educated with the asthma action plan. They were monitored for 2 to 3 months - the same survey distributed to evaluate the effectiveness of the action plan. This study was approved by King Abdullah International Medical Research Center, Jeddah, Saudi Arabia (KAIMRC) and the Institutional Review Board (IRB). The objectives of both parts of the study were explained to the patients and keeping their confidentiality was assured. All data were collected and stored confidentially and data was translated, analyzed and interpreted without any fabrication or signification and presented as honesty.

All survey answers were entered into an excel sheet. Afterward, simple descriptive statistics were used to summarize our data by using Statistical Package for the Social Sciences; version 22 (SPSS Inc., Chicago, IL). For qualitative data, frequency and percentage were used and for quantitative data, median and interquartile were used. McNemar’s test used to compare the scores difference between pre-intervention and post-intervention results. The significance level is considered if P <0.05 with a confidence interval (CI) of 95%.

## Results

Demography

The study was conducted with 363 randomly selected asthmatic patients attending NGHA asthma clinics in Jeddah. The sample population was taken from patients of various backgrounds and educational levels for both genders as long as they were above five years old. As for gender, there were 58% (209) males and 42% (154) females. The participants’ median age was 23 ± 25 years.

The first phase of the study

Patients’ Knowledge About Asthma Medications and Attack Management Questions

Regarding the patients’ adherence to their maintenance medication when they feel good and have no symptoms, only 46% (168) of the sample population were adherent to their maintenance medication and 88% (320) of patients were able to differentiate between maintenance and reliever medications. The patients’ confidence about their knowledge regarding the correct way to use their inhalers was as follow, 36% (131) patients were very confident, 56% (202) were slightly confident, while only 8% (30) of the sample population did not know how to use it at all. About the patients’ confidence about their ability to control their asthma attacks, 23% (84) were very confident, and 38% (138) were slightly confident. However, 39% (141) of the patients did not know how to control their asthma attacks.

Questions About the Impact of Asthma on Patients’ Quality of Life and Functional Limitations

During the past two months, 30% (110) of the sample population experienced some limitations in their physical activity because of their asthma symptoms more than once a week, and 70% (253) experienced limitations once or less than one time a week. 149 (41%) of patients had to be absent from work or school more than once a week because of their asthma. While 59% (214) of patients had to be absent only one or less than one time a week. Besides, asthma has affected the social life of 63% (227) of the patients by keeping them from attending social events or playing with children at a rate of more than twice a week. However, 37% (136) of patients experienced these social limitations twice or less per week. Furthermore, 47% (171) of the sample demonstrates that their asthma symptoms cause them to wake up at night or in the early morning more than twice a week. While 53% (192) demonstrated that twice or less per week.

Questions About Asthma Exacerbation Related Events Rates

The results show that during the past two months, 217 (60%) of the sample population need to use rescue medication to relieve their asthma symptoms two times or less per week, while 146 (40%) need to use rescue medication more than two times a week. Regarding the exacerbation rate, 173 (48%) of patients did not have any exacerbation during the past year and 190 (52%) of the sample population had one or more during the past year. Besides, over the past year, 170 (47%) of patients needed to visit the emergency department once or more. Moreover, hospitalization occurred in 98 (27%) once or more during the past year due to their asthma.

Patients’ Evaluation Regarding Their Asthma and Peak Expiratory Flow Values

The results of patients’ opinion about their overall evaluation of their asthma control during the past two months shows that 15 (4%) of the sample population had no control, 49 (13%) had poor control, 136 (38%) had moderate control, 122 (34%) had good control, and 41 (11%) had complete control. The peak flow meter was applicable only for eight patients out of 363 patients with the following values: 81, 510, 520, 520, 550, 610, 610, and 620.

The second phase of the study

Only 53% (192 patients) out of the 363 subjects, who were invited again after the first phase of the study, were willing and able to participate in the second phase. Out of those 192 subjects, 132 patients (69%) were considered as dropouts because they either failed to adhere to the action plan, or show the unwillingness to complete the study, or didn’t attend to the follow up sessions, or develop one of the exclusion criteria, or didn’t fill the post-action plan survey. The result of the rest of 60 patients (31%) who completed the whole study is presented in Table 1.

**Table 1:** The patients' results before and after following the written action plan

Questions	Pre-action plan n=60	Post-action plan n=60	P-value
Patients’ knowledge about asthma medications and attack management:			
1- When you are feeling well (i.e. free of asthma symptoms) do you always take your preventer inhaler?	66.1%	86.7%	0.002
2- Are you sure you know which is your reliever and which is your preventer inhaler?	74.6%	93.3%	>0.000
3- Are you confident that you can use your inhalers correctly?	62.7%	66.7%	0.839
4- Are you confident that you can control asthma exacerbation?	27.1%	50.0%	0.007
The impact of asthma on patients’ quality of life and functional limitations:			
5- During the past 2 months, how often did your asthma prevents you from doing physical activities at work, school or home? a) More than once a week b) Only once or less than one time a week	25.4% 74.6%	18.3% 81.7%	0.481
6- During the past 2 months, how often did your asthma prevents you from going to work, school or other daily activities? a) More than once a week b) Only once or less than one time a week	20.3% 79.7%	6.7% 93.3%	0.039
7- During the past 2 months, how often did your asthma prevents you from attending social events or plying with the other children? a) More than two times a week b) Two times or less a week	27.1% 72.9%	3.4% 96.6%	>0.000
8- During the past 2 months, how often did your asthma symptoms (wheezing, coughing, chest tightness, shortness of breath) wake you up at night or earlier than usual in the morning? a) More than two times a week b) Two times or less a week	42.4% 57.6%	20.3% 79.7%	0.011
asthma exacerbation related events rates			
9- During the past 2 months, how often have you used your reliever inhaler (Ventolin)? a) More than two times a week b) Two times or less a week	45.8% 54.2%	18.6% 81.4%	>0.000
10- During the past year, how often did you suffer from asthma exacerbation (wheezing, coughing, chest tightness, shortness of breath)? a) one or more during the past year b) Did not happened during the past year	78.0% 22.0%	35.6% 64.4%	>0.000
11- During the past year, how many times did you need to visit the emergency department because of your asthma? a) one or more during the past year b) Did not happen during the past year	61.0% 39.0%	6.8% 93.2%	>0.000
12- During the past year, how many times have you been admitted to the hospitals because of your asthma? a) one or more during the past year b) Did not happen during the past year	16.9% 83.1%	1.7% 98.3%	0.004
Patients’ evaluation regarding their asthma and peak expiratory flow values:			
13- during the past 2 months, how would you rate your asthma control? a) Complete or Good control b) Moderate control c) Poor or No control	47.5% 39.0% 13.6%	71.2% 27.1% 1.7%	0.023
14- Write your result of peak flow meter If applicable?	It was applicable only for one of the subjects with a pre result of 81 and a post result of 90.		

## Discussion

In terms of patients’ knowledge, the results showed that 38.8% of patients did not know how to control their asthma attacks. According to the current guidelines for the management of asthma, patient education and regular follow-up with healthcare professionals are important points to emphasize [11]. Therefore, we may link the lack of knowledge about asthma attacks control to the full patients’ dependence on hospitals, the absence of educational sessions and the crisis-oriented. Besides, more than half of the patients, about 195 (53.7% of the sample), were not adherent to their prophylactic medications when they feel good and have no symptoms. The American Journal of Respiratory and Critical Care Medicine supported this result as it concludes one of its studies by stating that, “A significant proportion of patients with difficult-to-control asthma remained not adherent to corticosteroid therapy” [12]. Through the interview, we noticed that most of the patients think that using this medication when they are in good condition is useless, and cause them addiction to this drug, or reduce their immunity. This result demonstrates the low awareness of the importance of the maintenance drugs among our patients and attracts the attention of the medical care systems to encourage and support the allocation of sufficient resources to permit barriers to self-management to be discussed and treatment plan negotiation [13].

According to the American Journal of Respiratory and Critical Care Medicine, “Negotiating patients' treatment decisions significantly improve adherence to asthma pharmacotherapy and clinical outcomes” [14]. Furthermore, results showed that almost all of the population do not have any idea about peak flow meter and its benefit in assessing asthma state and keeping the condition under control. Maybe the fact that the hospital does not make this device available for the concerned patients and that doctors do not focus on giving the necessary information about it, helped in this knowledge gap. The peak flow meter is very useful to asthmatic patients to start to follow their condition and be interactive with the follow-up and the treatment by themselves.

Some limitations occurred during the study. First of all, a group of patients tends to deny their disease even though they use asthma medications. Also, we distributed the questionnaire for 363 NGHA patients. However, most of the patients were not able to attend the asthma clinic due to long-distance between their homes and the clinic. So only 80 patients came to the clinic and received the asthma action plan and did the initial assessment. Moreover, 60 patients were followed up after three months of receiving the asthma action plan, while the rest did not show up for the post- asthma action plan assessment. Another limitation is that we did not analyze our results according to the severity of asthma and the patient’s confidence in managing their disease.

## Conclusions

The results of this survey demonstrate that our asthmatic patients do not have any written asthma management instructions and insufficient control levels with low exacerbation recognition techniques. Therefore, more efforts should be carried out to spread the use of written plans and involve asthmatic patients in the management of their disease. Moreover, and as reflected in our second part of the study, following asthma action plan helps in increasing the patients’ knowledge about their condition, improving their quality of life and functional limitations, and increasing the confidence level of patients about controlling their asthma. We recommend to expand the use of written asthma action plans as well as providing patients with their own peak flow meters after educating them about its importance.
